# Mind the gaps: investigating the cause of the current range disjunction in the Cape Platanna, *Xenopus gilli* (Anura: Pipidae)

**DOI:** 10.7717/peerj.166

**Published:** 2013-09-26

**Authors:** Deborah J. Fogell, Krystal A. Tolley, G. John Measey

**Affiliations:** 1 Applied Biodiversity Research Division, South African National Biodiversity Institute, Cape Town, South Africa; 2 Department of Zoology, University of Cape Town, Rondebosch, South Africa; 3 Department of Botany and Zoology, University of Stellenbosch, Matieland, South Africa; 4 DST-NRF Centre for Invasion Biology (CIB), Department of Zoology, Nelson Mandela Metropolitan University, Port Elizabeth, South Africa

**Keywords:** Hybridization, mtDNA, Morphometrics, Population genetics, Range disjunction, *Xenopus laevis*

## Abstract

Low-lying areas of the Cape at Africa’s south-westernmost tip have undergone dramatic marine-remodelling, with regular changes in sea-level following glacial cycles. Species for which marine barriers are impenetrable underwent concomitant radical distribution changes which may account for current range disjunctions. The Cape platanna, *Xenopus gilli*, is a frog distributed in only three disjunt areas within low-lying regions of the southwestern Cape. We determined the relationship between frogs from these three disjunct areas, by using a combination of morphometric analysis and mtDNA (ND2 and 16S fragments) sequences of 130 frogs from eight ponds. Coalescent analyses on molecular data dated the divergence in two major clades to around 4.6 Mya, a period during which major uplifting on the eastern side of the subcontinent caused climate changes throughout southern Africa. Principal components analysis showed significant morphometric differences between each clade on head and limb measurements. Consistent differences in ventral colouration and patterning were also observed. We report on increased levels of hybridisation with *X. laevis* throughout the range of *X. gilli*, which reaches at least 27% hybrids in some ponds. Urgent conservation actions are required to control habitat loss from alien invasive vegetation, and prevent introgression with the domestic-exotic, *X. laevis*.

## Introduction

Today, southern Africa is primarily a semi-arid region with subtropical conditions along the east coast (Tyson & Preston-Whyte, 2000). The southwest of the region, known as the “Cape”, is one of the world’s megadiverse areas, known for its floral diversity: specifically the Cape Floral Region made up of the Fynbos and Succulent Karroo biomes with around 70% endemism of over 9000 plant species (Rouget et al., 2003). However, elevated floral diversity of the fynbos biome is relatively recent, corresponding with the establishment of this biome during the late-Pliocene (∼5 Mya: Cowling & Pressey, 2001; Cowling, Proches & Partridge, 2009; Goldblatt, 1997; Linder, 2005). This radiation is attributed, in part, to substantial uplifting on the eastern side of the subcontinent leading to significant reduction of rainfall in the western area and aridification of the subcontinent’s interior (McCarthy & Rubidge, 2005; Partridge & Maud, 2000).

The high percentage of Cape floral endemics is associated with a wealth of faunal endemism in terrestrial, freshwater and marine ecosystems (Picker & Samways, 1996). Cladogenesis of faunal lineages in this region is thought to have been influenced by major shifts in climate, especially in the Pliocene (e.g., Daniels, Gouws & Crandall, 2006; Matthee & Flemming, 2002; Measey, Hopkins & Tolley, 2009; Price, Barker & Villet, 2007; Smit, Robinson & Van Vuuren, 2007; Tolley et al., 2006; Tolley, Chase & Forest, 2008). The area is also known for its rich amphibian fauna, particularly of range restricted and endemic species (Alexander et al., 2004; Angulo, Hoffmann & Measey, 2011; Holt et al., 2013; Poynton, 1964; Seymour et al., 2001), many of which are also of conservation concern (Angulo, Hoffmann & Measey, 2011; Botts, Erasmus & Alexander, 2012; Mokhatla et al., 2012; Stuart et al., 2008).

**Figure 1:**
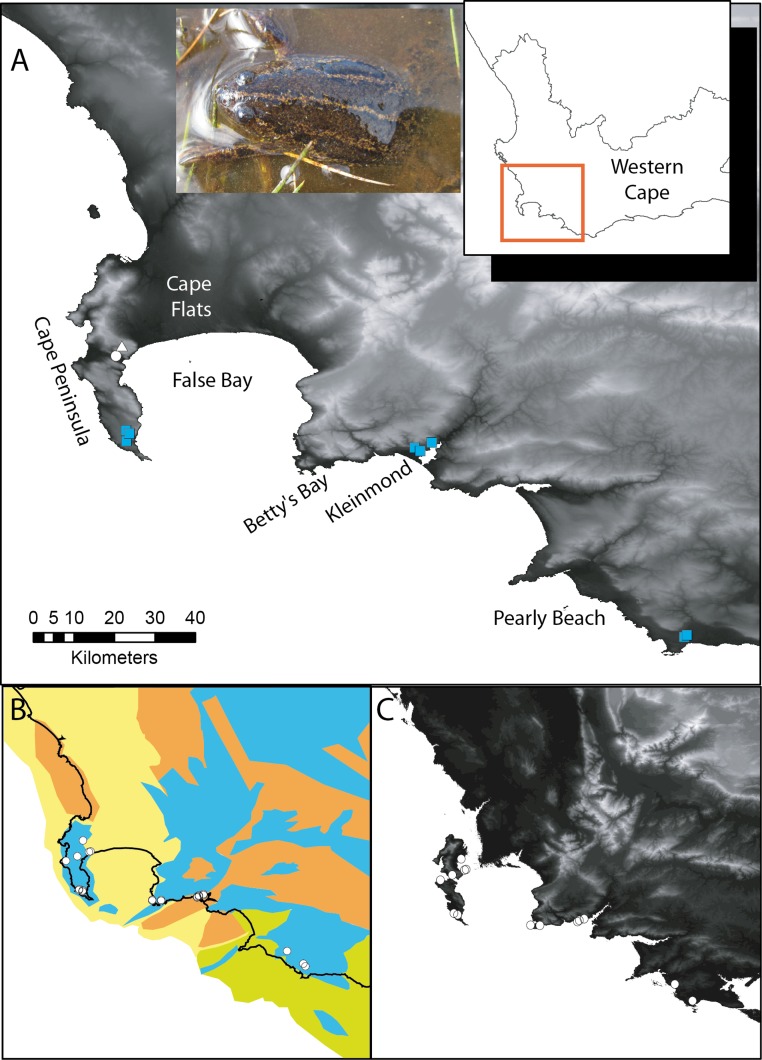
*Xenopus gilli* sites in the southwest of South Africa. (A) Sampling sites for the present study, squares indicate where *Xenopus gilli* was sampled for this study. A triangle denotes an introduced population (Measey & de Villiers, 2011), and the white filled circle the presumed type locality: Silvermine River. Note that the squares are distributed in three disjunct areas at the end of the Cape Peninsula near the towns of Kleinmond and Pearly Beach. (B) Vegetation types (after Mucina & Rutherford, 2006) and their projection onto the exposed Agulhas bank after a 120 m reduction in sea level (current coastline is shown as a black line) following Compton (2011). Note that current and historical sites for *X. gilli* (circles) are associated with lowland Sand-stone fynbos (blue) and not with Sand fynbos (yellow), Renosterveld (brown) or Strandveld (green). (C) Relief map of the same southwestern Cape region after a 25 m increase in sea level. Note that the Cape peninsula becomes two islands separated from the mainland by inundation of the Cape Flats. *X. gilli* sites in Betty’s Bay and Kleinmond are separated from those at Pearly Beach by high ground and a salt-water estuary.

The Cape platanna, *Xenopus gilli* Rose & Hewitt, is one of several species with a disjunct distribution on either side of False Bay (Fig. 1) (for other examples see Rourke, 1972; Swart, Tolley & Matthee, 2009), accompanying suggestions that past oceanic intrusion over the Cape Flats may have created a barrier to migration. However, *X. gilli* has three distinct areas of distribution (Fig. 1), prompting questions about the periodicity of disjunctions. Two other coastal lowland anuran species, *Amietophrynus pantherinus* (Smith) (the western leopard toad) and *Microbatrachella capensis* (Boulenger) (the micro frog), share similar distributions and restricted dispersal ability (Minter et al., 2004). A genetic study of *A. pantherinus* showed that populations on either side of the Cape Flats became disjunct, not as a result of anthropogenic habitat loss, but naturally as the area underwent significant drying during the Holocene (Measey & Tolley, 2011).

Evans et al. (1997) analysed the distribution, frequency and evolutionary relationships of *X. gilli* and revealed a deep split between distinct lineages on either side of False Bay. Further, they suggested that these clades had become isolated by oceanic transgression of the Cape Flats that occurred and persisted through the Pliocene and Pleistocene. Evans et al. (2004) estimated a phylogeny based on mtDNA of the genus *Xenopus* including single specimens of *X. gilli* from either side of False Bay (Cape Point and Betty’s Bay), and dated their divergence at 8.5 Mya (4.8–13.4). However, *X. gilli* is a fynbos endemic, specialising in the acid-blackwater pools that are characteristic in this vegetation type, and the fynbos biome is only thought to have become established in the early Plio- Pleistocene (see below). The dating and genetic distance between these clades is larger than between other species in the genus *Xenopus* (Evans et al., 2011; Evans et al., 2004), and it would be surprising if this was not accompanied by some morphological distinction. However, the genus *Xenopus* is rather conservative in its morphological variation such that species are often defined by their calls, colouration and markings (Kobel, Loumont & Tinsley, 1996).

As comprehensive sea-level models have been developed for glacial and interglacial periods (e.g., Raymo et al., 2011; Zachos et al., 2001), it is possible to test hypotheses relating to speciation and migration events across oceanic barriers to dispersal (Compton, 2011; Lambeck & Chappell, 2001). During the course of the Pliocene inundation of the Cape Flats that occurred approximately 3.4 to 4.5 million years ago, sea levels were between 25 to 40 m higher than present (Wardlaw & Quinn, 1991, Fig. 1C). Although incursions may have formed saltwater barriers, there have been subsequent glacial periods in which the oceans have regressed and the continental shelf around the southern tip of Africa has been exposed, allowing for the migration of organisms across False Bay (Compton, 2011, Fig. 1B). During these glacial events (36.5 to 13.8 Kya) the sea-level was up to 130 m lower than present, exposing the shallow Agulhas Bank and extending the furthest tip of the continent over 140 km southward (van Andel, 1989), opening portals of migration for many Cape lowland species (Compton, 2011; Schreiner, Roedder & Measey, 2013). Furthermore, studies of sea-level change have shown that, compared to present day levels, such regressions have been far more substantial than rises over the last 3 My (Bintanja & van de Wal, 2008).

The aim of this study is to determine existing differences between the three disjunct areas of occurrence for *X. gilli* (Fig. 1). Specifically we hypothesised that sea-level rise (or fall) brought about isolation which contributed to divergence in *Xenopus gilli*. We examined these hypotheses using both morphometric data and molecular markers of *X. gilli* from multiple sites within each of the three disjunct areas. We dated the divergence time across the geographic gaps using a coalescent approach with sequences from two mitochondrial genes. Lastly, we compared size-corrected morphometric data from each genetically defined population.

## Materials and Methods

### Study species and sampling

*Xenopus gilli*, the Cape platanna, inhabits lowland acid blackwater pools characteristic of the fynbos biome (Picker & de Villiers, 1989). Anthropogenic disturbances, intense urbanisation and the spread of alien vegetation has resulted in declines of populations of *X. gilli*, so that this species is now considered Endangered (SA-FRoG & IUCN SSC-ASG, 2011). *Xenopus gilli* was initially known from only two localities (the Cape Peninsula and adjoining Cape Flats) but in the 1970s a population was discovered approximately 50 km to the east, on the opposite side of False Bay (see Fig. 1A). Lastly, in 1987 a third disjunct cluster of sites was found near the town of Pearly Beach (Picker & de Villiers, 1989, Fig. 1). The Cape Flats were farmed for more than two centuries, and it is postulated that this resulted in the reduction of *X. gilli* populations (Kobel, Du Pasquier & Tinsley, 1981; Picker, 1985; Picker & de Villiers, 1989; Simmons, 1985). However, more recent urbanisation of the Cape Flats over the last 50 years (see Rebelo et al., 2011), would have extirpated any remaining populations. In addition to loss of habitat through land use change, *X. gilli* is threatened through introgression from its sister species, *Xenopus laevis* (Daudin) (see Picker & de Villiers, 1989) which is known for its invasive abilities on four continents (Measey et al., 2012). In South Africa, *X. laevis* is considered to be a domestic exotic, readily invading disturbed waters, and it is present throughout the entire range of *X. gilli* (Measey & Channing, 2003; Measey & Davies, 2011).

Morphological intermediates between these two *Xenopus* species from the Cape of Good Hope Nature Reserve (COGH: at the tip of the Cape peninsula: see Fig. 1) were first noted by Rau (1978). The severity of the threat of extensive introgression was questioned due to the low fitness and reproductive capability of any hybrid offspring that were produced (Picker, 1985), and later by the low frequency of genetically hybrid individuals (Evans et al., 1997). Although studies incorporating molecular genetics suggested that there was relatively little hybridization present between the two species (Evans et al., 1997; Evans et al., 1998), the threat posed by *X. laevis* remains of conservation concern (Measey, 2011; SA-FRoG & IUCN SSC-ASG, 2011).

*Xenopus gilli* was first described from the Silvermine River (Rose & Hewitt, 1927) and is usually characterised by a venter marked with bright yellow-orange and black mottling; although this is more distinct in some animals than others (de Villiers, 2004). Dorsally, *X. gilli* is yellow-brown or beige with large paravertebral dark brown stripes extending from the eyes, down the length of the body and breaking up into patches on the lower back and legs. They exhibit sexual size dimorphism with the larger females attaining a snout-vent length of approximately 60 mm and having distinct cloacal folds (de Villiers, 2004; Kobel, Loumont & Tinsley, 1996).

For this study, netted funnel traps baited with liver were set overnight and retrieved in the early morning on two consecutive days of each field excursion (see Measey, 2001), from March 2010 to August 2011. In addition to traps, seine nets were used at some localities during operations to remove *X. laevis* (see Measey & Davies, 2011). Collections of *Xenopus* were made at eight ponds across the three different areas. Three ponds were sampled on the Cape peninsula (in the Cape of Good Hope Nature Reserve: CoGH), all within an area of 2 km^2^. All other collection sites were on the east side of False Bay (Fig. 1A) at ponds where *X. gilli* historically occurred (see Picker & de Villiers, 1989). *Xenopus gilli* were captured near Kleinmond (three ponds; hereafter Kleinmond) and near Pearly Beach (two ponds; hereafter Pearly Beach). We identified individuals as *X. gilli* from dorsal and ventral patterning, shape of head, and overall size (de Villiers, 2004; Kobel, Loumont & Tinsley, 1996). *Xenopus laevis* and suspected *X. gilli-laevis* hybrids were not included in our analyses.

Each *X. gilli* captured had the second phalange of the right hind foot clipped, taking care to not break the webbing between the toes. Blades were sterilised with 10% bleach solution between each procedure to prevent any spread of disease. Tissue samples for DNA analysis were stored in vials of molecular grade ethanol.

Permits were obtained from Cape Nature (permit number: AAA004-00322-0035) and South African National Parks (permit: Agulhas and Table Mountain National Parks, issued 21 July 2010). Ethics clearance was obtained from South African National Biodiversity Institute ethical clearance (number: 001/10).

### Morphometrics

All *Xenopus gilli* captured had a standard set of 14 measurements taken: snout-vent length (SVL), head length (HL), head width (HW), head height (HH), lower jaw length (LJL), ilial length (IL), ilial width (IW), femur (FM), tibia (TB), metatarsal (MT), longest toe (LT) on the hind limb, humerus (HM), radius (RD), and longest finger (LF) on the forelimb (see Herrel et al., 2012). All measurements were taken by DJF with digital callipers (with 0.01 mm accuracy) on the left side of each animal. In addition, images were taken of the dorsum and venter of each animal on scaled grid paper to avoid measuring animals recaptured.

### Genetics

Total genomic DNA was extracted from the tissue samples using a standard salt extraction procedure (Bruford et al., 1992) and fragments of two mitochondrial (mt) markers (ND2 and 16S) were amplified. However, as mtDNA is non-recombinant and is maternally inherited (Lamb & Avise, 1986) our analysis is limited to inferences based on the maternal lineage, specifically with regards to identifying any hybridization with *X. laevis*. The ND2 marker was amplified from vMet3 to vTrp (Cunningham & Cherry, 2004) and 896 base pairs were used for analysis (mtDNA BP 5980 to 6876, Roe et al., 1985). Polymerase chain reaction (PCR) annealing temperature was optimised at 57.1°C for 40 cycles and magnesium concentration was 3 mM MgCl_2_. The 16S marker was amplified from primers 16Sa to 16Sb (Palumbi, 1996) of which 517bp were used for analysis (mtDNA BP 4052 to 4559, Roe et al., 1985). PCR annealing temperature was optimised at 48.1°C for 40 cycles and magnesium concentration was 2.5 mM MgCl_2_. Electrophoresis was used to examine the quality of PCR products on a 2% agarose gel stained with Goldview and the high quality products were sent to Macrogen Inc. (Seoul, Korea) for sequencing.

### Data analysis

Geneious Pro 5.0.4 was used to edit and align the DNA sequences from each sample using a global alignment (Drummond et al., 2007) and the alignment was used to define haplotypes at each site. One animal from Cape Point was removed from all further analyses as it fell into an eastern haplotype population. Similarly, Evans et al. (1997) found another such animal in their study and speculated that it may have been mistakenly transported and introduced. Any other individuals that closely matched *X. laevis* (i.e., had >98% alignment with the ND2 region of EMBL accession HM991335) were considered to be hybrids and therefore removed from further analyses.

In order to determine whether sampling sites clustered on a population level, a spatial analysis of variance (SAMOVA) was conducted (Dupanloup, Schneider & Excoffier, 2002), using ND2 haplotype data and geographic co-ordinates of each of the 9 sample sites. The SAMOVA was run for *K* = 2–9 putative populations to determine the maximum *F_CT_* value, the highest proportion of differences between populations due to genetic variation (Dupanloup, Schneider & Excoffier, 2002). The results of the SAMOVA allowed for the consolidation of the DNA data into a Cape peninsula population and a combined Kleinmond and Pearly Beach population for further genetic and morphometric analyses (henceforth combined and referred to as the “eastern population”). Network 4.5.1.6 (Bandelt, Forster & Röhl, 1999) was used to consolidate the haplotype data into a Median-joining network. Arlequin 3.5 (Excoffier & Lischer, 2010) was used to provide a quantitative measure of haplotype and nucleotide diversity within localities. Sequence divergence between populations was estimated with uncorrected net *p*-distances using MEGA v. 5 (Tamura et al., 2011).

To examine whether any population had experienced historical demographic changes, Tajima’s D and Fu’s *F_S_* was used to test for departures from mutation–drift equilibrium (Chuang & Lee, 1997; Schneider & Excoffier, 1999) using Arlequin 3.5 (Excoffier & Lischer, 2010). For populations out of equilibrium, we then applied a model of demographic expansion (Excoffier & Lischer, 2010; Rogers, 1995; Rogers & Harpending, 1992). For populations that showed signs of expansion, we estimated the timing of the demographic shift; *t* = τ/(2*u*), where *t* is time in generations, τ is the age of the expansion in mutational units (estimated in the model of demographic expansion) and *u* is the sum of the per nucleotide mutation rate for the region sequenced (Rogers, 1995; Rogers & Harpending, 1992). For the present study, generation time was estimated at 2 years (de Villiers, 2004) and *u* was obtained from the same estimate of mutation rate as described for coalescence analysis (below).

Coalescent analysis was used to investigate time of divergence (*t*) between all the populations identified in SAMOVA (Cape Peninsula vs. eastern), using IMa2 (Hey, 2010). IMa2 runs Markov chain Monte Carlo (MCMC) simulations using a likelihood based analysis, under a Felsenstein framework (Hey & Nielsen, 2007). MCMC included a burn-in duration of 3 million steps, 10 000 genealogies saved and a geometric heating model with 20 chains, 0.96 as first and 0.9 as second chain heating parameters. A geometric heating scheme was applied to ensure sufficient rates of chain swapping to speed up the calculation process. The mutation rates for the ND2 and 16S regions of the mitochondrion were determined using the estimated time of divergence reported for the dated time of divergence between the two *X. gilli* lineages (8.5 Mya) in Evans et al. (2004). Mutation rate was estimated to be 0.52% change per lineage per million years, and compares favourably with other mutation rates published for ND2 (e.g., 0.69% Macey et al., 1998). For 16S, we used the same calculation to give us a rate of 0.10% change per lineage per million years, which is in the same general range as other published rates for this marker (e.g., 0.15% Vences et al., 2005). The software IMa2 uses the geometric mean of all mutation rates with upper and lower confidence intervals which were calculated from the upper and lower divergence bounds for *X. gilli*: 4.8 and 13.4 Mya (Evans et al., 2004). Preliminary runs in IMa2 were used to establish settings for priors, ensure adequate mixing and sufficiently high effective sample size (see Hey, 2010), this included setting migration to 0 between CoGH and other populations due to an absence of shared haplotypes between populations. Three final duplicate runs were made with the same parameters using IMa2 at Cornell University via internet upload (http://cbsuapps.tc.cornell.edu/IMa.aspx).

For the morphological analysis, all measurements were Log_10_-transformed to meet assumptions of homoscedascity, and then residuals were estimated for 13 variables against SVL to correct for size because amphibians are known to have indeterminate growth and thus their size positively correlates with age (Halliday & Verrell, 1988), including species in the genus *Xenopus* (Measey, 2001). To examine sexual dimorphism, a multivariate analysis of variance (MANOVA) was performed in the base package of R (R Development Core Team, 2011) using the residuals of morphometric data, separately for each genetically defined population (Cape peninsula and eastern). In addition, we checked for sexual differences within populations by using the locality and sex as an interaction term.

In the absence of sexual dimorphism for the variables measured (see Results), data from both sexes were pooled for all further analyses. To examine population level differences in morphology, a multivariate approach was taken. Size corrected residuals from the Cape peninsula (*n* = 50) and from the eastern population (*n* = 59) were used as input variables for a principal components analysis (PCA) to reduce the 13 morphometric variables into linear combinations of correlated variables (principal components-PCs). The Keiser-Meyer-Olkin (KMO) measure of sampling adequacy was applied to determine whether the dataset was adequate to conduct a PCA. The analysis included a varimax rotation to minimise the number of variables with high loadings on each PC, which simplifies the interpretation of the PCs extracted. Variables with communalities greater than 0.5 were retained in the analysis and only PCs with eigenvalues greater than 1.0 were extracted. The principal component scores were saved, and ANOVA was carried out on the PCs extracted using R (R Development Core Team, 2011).

## Results

A total of 130 frogs, which were identified as *Xenopus gilli*, were sampled from eight ponds located within in each of three geographic areas: Cape peninsula, Kleinmond and Pearly Beach (Fig. 1A). On the Cape peninsula, adult *X. laevis* were captured in two of the three ponds sampled, including one which was isolated by a concrete wall (see Picker & de Villiers, 1989). All *X. laevis* captured at the Cape of Good Hope were removed as a part of a South African National Parks initiative to safe guard remaining *X. gilli* (see Measey & Davies, 2011). In the eastern sites, at Kleinmond, one pond contained only *X. laevis* and *X. gilli-laevis* hybrids, while a second contained a majority of hybrids, so no individuals from these two sites were used in this study. Thus, individuals from only two ponds near Kleinmond, where all animals appeared to be *X. gilli*, were used, plus all frogs from two ponds near Pearly Beach, as no morphological hybrids were observed there.

### Genetics

Sequences of 896 base pair fragment of ND2 were obtained from 128 individuals identified in the field as *X. gilli*, plus 517 bp of 16S from a subset of 14 animals. Of the 39 *X. gilli* collected and sequenced for ND2 from the Cape peninsula, one was found to match a GenBank *X. laevis* sequence. From the 48 sequenced animals from Pearly Beach, four ND2 haplotypes matched *X. laevis*, but for the Kleinmond sample, this proportion reached 27% of 41 animals sequenced (Table 1). All animals with *X. gilli* appearance, but with *X. laevis* haplotypes were subsequently removed from genetic and morphometric analysis. Spatial clustering analysis (implemented in SAMOVA) unambiguously showed that the nine sampling sites could be grouped into two populations, corresponding to animals collected in the Cape peninsula (western) and combined eastern sites (Kleinmond and Pearly Beach: FST = 0.985; *P* < 0.001). For 16S, all four individuals sequenced from the Cape peninsula were of a single haplotype identical to *X. gilli* from Cape Point sequenced by Evans et al. (2004, AY581649). Of ten animals sequenced from the eastern region, eight were identical to an *X. gilli* previously sequenced from Betty’s Bay by Evans et al., (2004, AY581650), while two more individuals (both from Kleinmond) had a single base pair change. Between the Cape peninsula (within 0.00% divergence) and eastern sites (within 0.069% divergence), the net *p*-distance for 16S was found to be 1.7%.

**Table 1:** Sample sizes for genetic (*N*) and morphometric samples of the Cape platanna (*Xenopus gilli*) in the Cape peninsula, Kleinmond and Pearly Beach disjunct areas together with their EMBL accession numbers.

Site group	Gene fragment	*N*	Haplotypes	EMBL accession number	Morphological sample size
Cape Peninsula	ND2	38	3	HG427452-4	50
	16S	4	1	HG427449	
Kleinmond	ND2	30	13	HG427464, HG427467, HG427471, HG427474-82, HG427484	21
	16S	6	3	HG427450-1	
Pearly Beach	ND2	44	24	HG427455, HG427456, HG427457, HG427458, HG427459, HG427460, HG427461, HG427462, HG427463, HG427465, HG427466, HG427468, HG427469, HG427470, HG427471, HG427472, HG427473, HG427475, HG427476, HG427477, HG427478, HG427480, HG427481, HG427483	38
	16S	4	1	HG427450	

**Figure 2:**
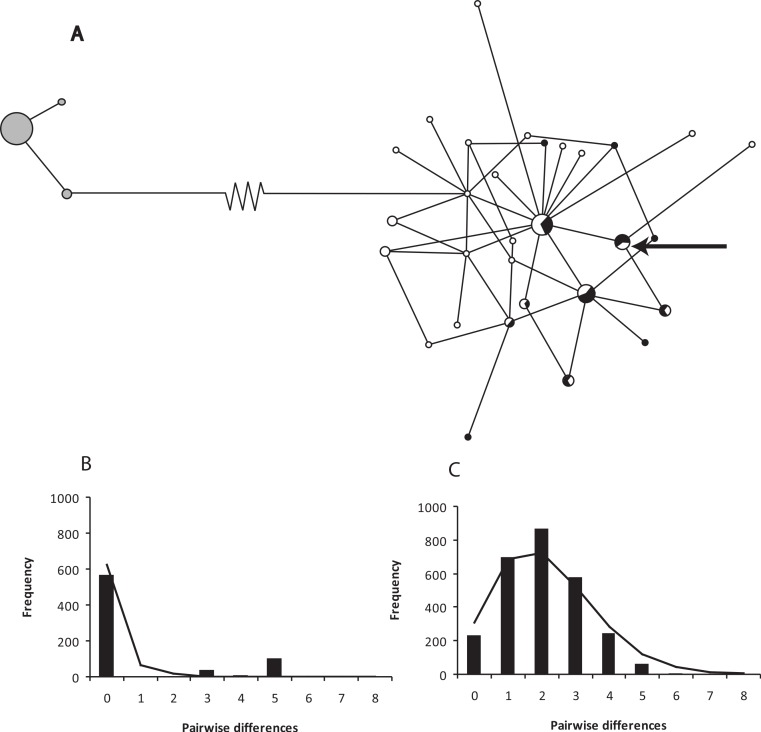
A network of ND2 haplotypes for *Xenopus gilli* populations. (A) Median-joining network diagrams of the number and structure of *X. gilli* haplotypes in the Cape peninsula and eastern populations based on ND2. Cape peninsula animals are indicated in grey, Pearly Beach indicated in white and Kleinmond in black. Circles are proportional to the number of individuals sequenced belonging to each haplotype and lines represent the number of nucleotide base pair changes from one to the other. Note that the 74 base pair changes between the two haplotype groups are represented by a broken line. The arrow points at a haplotype not included in the analysis but found on the Cape peninsula. (B) Pairwise difference and frequency for ND2 haplotypes for *Xenopus gilli* from the Cape peninsula, and (C) Pearly Beach and Kleinmond.

The haplotype network for ND2 shows two clear groups separated by 74 base pair changes (Fig. 2). Sequence divergence between populations shows a net *p*-distance of 8.8% at the ND2 locus between animals from the Cape peninsula and the eastern population. The Cape peninsula had only three ND2 haplotypes amongst 38 individuals (*p*-distance within = 0.25%), while the 74 animals sequenced from the eastern population share 30 different haplotypes (*p*-distance within = 5.63%).

The eastern *X. gilli* populations had a higher ND2 nucleotide and haplotype diversity than animals from the Cape peninsula (Table 2). However, the 95% confidence intervals show that the Cape peninsula population is significantly less diverse for haplotype diversity (*h*), but not nucleotide diversity (π). The low haplotype diversity on the Cape peninsula is also demonstrated by the haplotype network (Fig. 2A). Both Tajima’s D and Fu’s F test suggest that the eastern population is out of mutation-drift equilibrium (Tajima’s *D* = −1.6, *p* = 0.032; Fu’s *F_S_* = −27.2, *p* < 0.001), but this was not detectable within the Cape peninsula. The model of demographic expansion fitted the observed distribution for the eastern population (SSD = 0.005, *p* = 0.08) suggesting the population has undergone a demographic shift. The value obtained for τ = 2.16 (95% CI: 1.29–2.78), provided an estimate for years since population expansion (*t*) of 75.7 thousand generations or ca. 151.4 Kya.

**Table 2:** The gene diversity (*h*) and nucleotide diversity (π) within sites as well as the tests of neutrality to indicate population stability where *p* < 0.05 is considered significant.

Population (*n*)	*t*	SSD (*p*)	*R*(*p*)	Fu’s *F*(*p*)	*D*(*p*)	*H*	*h*	π
East (74)	2.2	0.005 (0.11)	0.07 (0.08)	−27.2 (<0.001)	−1.59 (0.02)	30	0.91 (±0.020)	0.002 (±0.0014)
							0.89–0.93	0.0006–0.0034
West (38)	3.0	0.040 (0.02)	0.68 (0.69)	1.52 (0.17)	−1.02 (0.81)	3	0.20 (±0.083) 0.117–0.283	0.001 (±0.0008) 0.0002–0.0018

**Figure 3:**
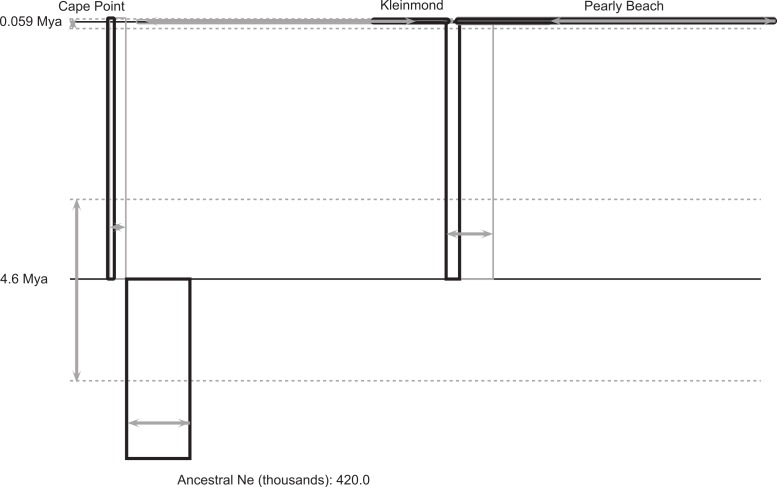
IMa2 coalescent analysis of *Xenopus gilli*. Result of the coalescent analysis (in IMa2) provides histories for the three disjunct areas sampled which are represented as boxes (for sampled and ancestral populations), horizontal lines (for splitting times). The size of the boxes on the horizontal axis is proportional to estimated current and ancestral population sizes. Time is represented on the vertical axis, with the sampled sites at the top representing the sampling time. Estimated error is shown in grey (boxes and arrows).

The coalescent analysis provided an estimated divergence time between the Cape peninsula and eastern population of ca. 4.6 Mya with a confidence interval from 3.2 to 6.4 Mya (Table 2; Fig. 3), which falls entirely within the Pliocene.

### Morphometrics

Morphometric measures were made on a total of 109 individuals from all sites. Following the results of SAMOVA analysis we conducted morphometric tests on animals collected from all eastern populations (Pearly Beach and Kleinmond; *n* = 59) compared to those from the Cape peninsula (*n* = 50). The first test for sexual size dimorphism confirmed that in both populations females were significantly larger than males by around 25%: Cape Peninsula females had a mean SVL of 43.0 mm (SE 0.752), while males’ was 34.6 mm (SE 0.528; *t*_1,48_ = −7.248; *P* < 0.001). For the eastern population, mean female SVL was 44.2 mm (SE 0.071), with males’ mean of 34.3 mm (SE 0.618; *t*_1,57_ = −8.88; *P* < 0.001). No significant differences were found overall between sexes using size corrected measurements in a MANOVA (Pillai = 0.1250, *P* = 0.42). Additionally, there was no significant difference for the interactive term: sex and population (Pillai = 0.1692, *P* = 0.15), indicating that within populations, the sexes did not differ in their morphology. Thus, for all further tests sexes were pooled.

**Figure 4:**
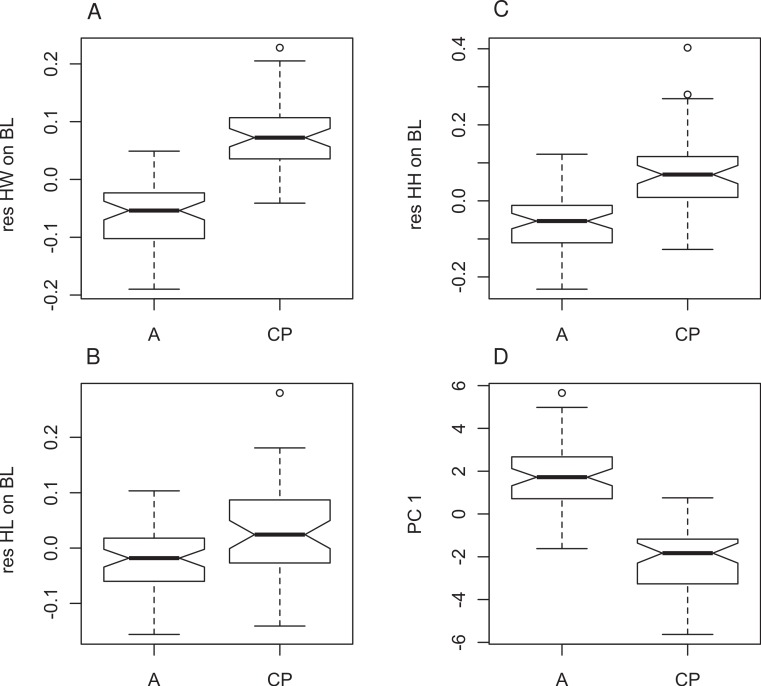
Morphometric differences between genetically defined populations of *Xenopus gilli*. Notched box and whisker plots display the direction of significantly different head measurements between sites identified by genetic differences (CP: Cape peninsula and (A) Agulhas plain sites Pearly Beach and Kleinmond). All plots are corrected for size and therefore based on residuals of each measurement on body length (SVL). (A) Head length (HL), (B) head width (HW) and (C) head height (HH) are all significantly different between sites (*p* < 0.001). (D) Values for principle component 1 also show significant differences. When notches do not overlap, there is ‘strong evidence’ that their medians differ (Chambers et al., 1983).

Four principal components (PCs) were extracted in the PCA, which accounted for 73.3% of the total variation in the dataset (Table 3). The KMO test indicated sampling was adequate (KMO = 0.83), all communalities were high suggesting that all variables were reliable contributors to the analysis. The four PCs extracted (Table 3) loaded highest with measures of the limbs and head (metatarsal, head width, femur, ilial width, tibia, longest toe and head height in descending order) on PC1 (Fig. 4), and arm and ilial length (radius, ilial length, longest finger) on PC2. An analysis of variance (ANOVA) on the principal components showed significant differences between the populations for PC1 only (PC1 *F*_107,1_ = 180; *P* < 0.0001, PC2 *F*_107,1_ = 1.503, *P* = 0.2228; Fig. 4D) suggesting that they can be distinguished on the basis of limb dimensions and head height.

**Table 3:** Principle Components Analysis on morphometric measurements of *Xenopus gilli* corrected for size by residuals on body length (SVL). Only the first four principle components (PC1 to 4) are shown as they had Eigenvalues greater than 1. Variables are arranged by their contribution to each PC with significant contributing variables in bold.

Variable	PC1 (43.6%)	PC2 (12.0%)	PC3 (9.8%)	PC4 (7.9%)
Metatarsal	−**0.3740**	0.0195	−0.0968	0.0254
Head width	−**0.3713**	−0.0995	−0.0264	−0.1961
Femur	−**0.3547**	−0.0813	−0.1710	0.0236
Ilial width	−**0.3334**	−0.1801	0.0956	−0.1040
Tibia	−**0.3293**	0.2585	−0.1963	0.2572
Longest-toe	−**0.3266**	0.0333	−0.2983	0.0351
Head height	−**0.3143**	−0.2046	0.1633	−0.3047
Radius	−0.0375	**0.6008**	0.1352	−0.0999
Ilial length	−0.1778	−**0.4000**	0.3709	0.3488
Longest finger	−0.2243	**0.3144**	−0.2394	−0.2977
Humerus	−0.0656	0.3582	**0.6032**	−0.2495
Head length	−0.2370	−0.0371	**0.4506**	−0.0179
Lower jaw length	−0.1662	0.3022	0.1313	**0.7116**

## Discussion

There is considerable genetic and morphological variation between the two identified populations of *Xenopus gilli* distributed on either side of False Bay. However, for the two disjunct clusters of sites in the East (Kleinmond and Pearly Beach), our analyses did not suggest that they represent separate populations. Despite the disturbance to population distributions caused by sea-level change, our results from coalescent analysis of the deep genetic division between the two populations, dates back to 4.63 Mya (3.17 to 6.38 Mya). This is considerably younger than the estimate provided by Evans et al. (2004, 8.5 Mya, although it is close to their lower 95% confidence interval: 4.8 to 13.4 Mya). If standard mutation rates from the literature are applied (see above) a similar date of 3.44 Mya, with confidence intervals of 2.34 to 4.69 Mya is obtained. Variation in dates may relate to differences in mutation rates and the use of the geometric mean of mutation rates in IMa2, we therefore advise caution in ascribing certainty to exact dates. We do however, suggest that taken together, the best estimate is that divergence was within the Pliocene.

The original description of *X. gilli* by Rose & Hewitt (1927) was presumably from the same population that now resides on the Cape peninsula (see Fig. 1). These authors referred to head shape and size as defining characters in separating *X. gilli* from *X. laevis*. Relative to their size, the Cape peninsula animals had significantly broader and higher heads than those from the eastern population. In addition, animals from the eastern population had relatively shorter legs. These characters may relate to differences in performance between animals from the different habitat types (see Herrel & Bonneaud, 2012). On the Cape peninsula, natural water bodies are seasonally inundated wetlands, while eastern sites have typically larger, more permanent water bodies. We acknowledge that although our findings show no obvious sexual dimorphism, examination of a larger dataset should be conducted to confirm this result.

In addition to the quantitative morphometric study, a retrospective qualitative examination of the markings of each population revealed some consistent differences. There were no apparent differences between the animals with regards to their dorsal markings, both populations having large paravertebral dark brown stripes extending from the eyes, down the length of the body. However, the ventral surfaces were different with respect to positioning of the lateral line ‘stitch’ marks; these were more lateral in animals caught on the Cape peninsula, while those collected in eastern sites were more ventral. For eastern animals, the intensity of the yellow-brown and black marbling was much less apparent although some animals were more brightly coloured than others. Animals from the east also had transparent webbing between the toes on their hind feet, without exception, while the Cape peninsula animals had a continuation of their pigmentation from hind legs to webbing. As colour is often a plastic character, some of this ventral variation may be attributed to either a difference in water colour or carotenoids present in the chromatophores due to dietary differences (Kobel, Loumont & Tinsley, 1996). Both the colour and pattern differences, especially with regards to the lateral line organs should be quantified in future research.

### Historical events

Inundation of the Cape Flats was previously speculated to have caused the deep genetic division in the Cape *Xenopus* lineage (Evans et al., 1997), but repeated inundations both pre- and post-date our estimated divergence time (Compton, 2011), and so it seems unlikely that sea-level changes can be the sole factor responsible for the divergence. Sea-level change may have produced two different scenarios for these two *Xenopus* lineages: sea-level rises which contracted populations and sea-level falls which may have allowed a range extension and migration (cf Schreiner, Roedder & Measey, 2013). Furthermore, we suggest that the effects of climate change from 5 Mya are of key importance, when substantial uplifting led to significant reduction of rainfall in this area (McCarthy & Rubidge, 2005), reduction in temperature (Zachos et al., 2001) and a massive expansion in regional floristic diversity (Cowling, Proches & Partridge, 2009).

The Agulhas Bank was last exposed as recently as the last glacial maximum, some 20 Kya with a sea-level fall of around 140 m (Compton, 2011). The change in climate and land area since then is predicted to have had a major reduction on the distributions of Cape frogs (Schreiner, Roedder & Measey, 2013). At the last glacial maximum, much of the exposed land would have been sand fynbos vegetated dunes similar to that seen, and not occupied by *X. gilli*, on the Cape Flats today (see Compton, 2004). East of False Bay, the extension of the rivers across the exposed southern coastal plain would have in part coincided with bedrock supporting both Renosterveld and lowland sand-stone fynbos, vegetation types on which *X. gilli* are found today (Fig. 1B). On the Cape peninsula, it seems that only a small amount of land to the West and South would have been exposed, and this would also have been the currently uninhabited sand fynbos. Due East of the Cape peninsula (5–10 km) lies a large (approximately 50 km^2^) outcrop of sandstone which may have been able to support a population of *X. gilli*. It is not known whether *X. gilli* would have been capable of movement across the intermediary habitat, and there is a similar distance from this outcrop to the habitat extensions in the east. If dispersal was possible over these distances, there is the potential for movement of individuals across False Bay at each glacial maximum. Similarly, Rourke (1972) speculated about the dispersal of Proteacae across the same bridging habitat.

Our molecular data does not exclude movement between these lineages, as we did find a single individual in the Cape peninsula with a haplotype from the western population, as did Evans et al. (1998 see below). If single animals are capable of dispersal on this scale, our interpretation that this resulted from the release of captive animals would be erroneous. We suggest that in the east, as continuous appropriate habitat would have been uncovered by the retreat of the sea during the last glacial maximum (Fig. 1B), that this *Xenopus* lineage would have had a larger distribution. This may explain the star-shaped haplotype network (Fig. 2), and the significant results of neutrality tests, which suggest that this lineage has undergone recent expansion. Using mutation rates already described for ND2, we date this expansion to around 150 Kya, a period when the Agulhas bank was exposed with sea-level some 140 m lower than today (Compton, 2011).

Our approximate dates for divergence between the populations coincide with a period when sea-level is hypothesised to have been around 25 m higher (also see Raymo et al., 2011). However, unless this was the first marine incursion in the area, there is no reason to think that the substrate immediately prior (or after) to this time was any more suitable than it is today. Certainly the sea also covered the Cape Flats 1.5 Mya (Compton, 2004), and was even 20 m higher than current levels around 130 Kya (Compton, 2011). Thus, if sea-level has repeatedly covered the Cape Flats and uncovered the Agulhas Bank throughout the Pleistocene period, we look to other explanations for the continued separation of these lineages.

We consider that composition of the underlying substrate may be more likely to have had a lasting effect on the distribution of these acidophilic species. Distribution records for *X. gilli* suggest that this species is not tolerant of alkaline substrates, such as those currently in place over much of the Cape Flats area today (see Fig. 1B, Picker & de Villiers, 1989). The alkalinity of this area is thought to be in part due to repeated marine incursions, but with additional calcareous wind-blown sands resulting from the erosion of uplifted material (Compton, 2004; Cowling, Proches & Partridge, 2009). This suggests that areas that had been suitable for the ancestor of these populations were continually reduced over the last 5 My.

Climatic change also followed this period of substantial uplifting on the eastern side of the subcontinent and is thought to have led to significant reductions of rainfall in the west and aridification of the interior of South Africa (McCarthy & Rubidge, 2005; Partridge & Maud, 2000). The start of these substantial changes, around 5 Mya, coincides with our estimated split within *Xenopus gilli* and the rapid diversification and establishment of the fynbos biome ca. 3–5 Mya (Cowling & Pressey, 2001; Goldblatt, 1997), on whose acid-blackwaters *X. gilli* now depends (Picker, 1985). This same period is known to have resulted in radiations of other faunal groups in this specific area of the south-western Cape (e.g., Smit, Robinson & Van Vuuren, 2007; Swart, Tolley & Matthee, 2009; Tolley, Chase & Forest, 2008; Tolley et al., 2010). Thus, the tectonic uplift began a series of processes (spread of alkaline substrate and drying) which reduced the range of the most recent common ancestor of these distinct lineages, eventually leading to their divergence around 4.63 Mya. Further, we postulate that these processes, which have been ongoing, continue to reduce the ranges of these lineages (and other species such as *Microbatrachella capensis*), and this might explain why there was not a more recent genetic expansion during the last glacial maximum. Most recently however, anthropogenic factors have further reduced the ranges of these *X. gilli* populations, raising concern for the conservation of these species.

### Conservation

On the Cape peninsula, there has been a reduction in the known range of *Xenopus gilli* over the past century. The type series was said to be collected from the Silvermine River and adjacent Cape Flats (Rose & Hewitt, 1927), and in the same publication, it was noted that frogs collected from these localities were already outnumbered by *X. laevis* (see also de Villiers, 2004). The last record of this species outside of the CoGH is a specimen collected at Zeekovlei in the mid-1970s (in the Iziko Museum, Cape Town: Picker & de Villiers, 1989). This dramatic and rapid reduction in range may in part explain the low haplotype diversity observed in population sampled from the Cape peninsula (see also Cressey, Measey & Tolley, in press). The disparity between haplotype (*h*) and nucleotide (π) diversity between the two lineages is indicative of a bottleneck in the Cape peninsula, with a reduction in the number but not in the diversity of the remaining haplotypes. The distribution of pairwise differences between haplotypes (Fig. 2B) is also suggestive that intermediary haplotypes have been lost. The scenario is similar to that described in other historically documented cases of population bottlenecks (Hoelzel et al., 2002; Weber et al., 2000).

Natural hybridisation between *Xenopus laevis* and *X. gilli* was reported as far back as the 1970s (Kobel, Du Pasquier & Tinsley, 1981; Picker, 1985; Picker & Samways, 1996; Rau, 1978; Simmons, 1985), although continuing investigations showed that hybrid males were not fertile, and hybrid females had a reduced fecundity (Gurdon, 1996). The first genetic investigations into the frequency of hybrids found only a single (nuclear) hybrid in 32 *X. gilli* (and 38 *X. laevis*) sampled in the eastern area, while none were found from 59 sampled from the CoGH reserve (Evans et al., 1998). Thus, these authors concluded that introgression was not the greatest obstacle to *X. gilli* conservation. Our study, sequencing mtDNA only, avoided sampling any obvious *X. laevis* or putative *X. laevis-gilli* hybrids, but still found substantial numbers of animals which were morphologically identified as *X. gilli* (see methods for characters) but result from a *X. laevis* maternal lineage. From relatively low levels (2.5%) on the Cape peninsula, to 27% in ponds near Kleinmond where *X. laevis* and hybrids have come to dominate some historically known *X. gilli* sites. Ponds near Pearly Beach, which were previously thought to contain only pure *X. gilli* (see Evans et al., 1998; Picker & de Villiers, 1989), were found to have 8% hybrids, even though no *X. laevis*-like animals were seen. Our results suggest that hybridisation has greatly increased in both lineages and this aspect requires better investigation using nuclear markers and larger sample sizes.

Although we decided to remove the single individual found in CoGH with the eastern haplotype from our analysis, there remains the question surrounding the provenance of this individual with a haplotype also found by Evans et al. (1997). There are two possibilities: first that this individual was anthropogenically introduced into CoGH, or second that it represents ancient migration from the eastern population to the west. We reason that if it was possible for animals to migrate east to west, then the return route should also have been possible, and we might therefore expect to see instances of peninsula haplotypes in eastern populations. Given that the last time that the Cape Flats were covered was some 1.5 Mya (Compton, 2004), migrants from that time should be discernible from modern movements through further genetic testing such as micro-satellites. It is important to conduct such a test as otherwise these animals present another introgression threat to the remaining population on the peninsula.

Lastly, we suggest that the eastern lineage of *X. gilli* requires taxonomic attention through data on calls, patterning and lateral line configuration. Despite recent conservation work at CoGH removing hundreds of *Xenopus laevis*, hybridisation poses an ongoing threat (see Measey & Davies, 2011). The eastern population occurs over a much wider area, but all of this is threatened by invasive alien plants (as well as hybridising *X. laevis*). Despite good and clear documentation on the threats to this species, all appear to continue unabated.

## Additional Information and Declarations

### Field Study Permissions

The following information was supplied relating to ethical approvals (i.e., approving body and any reference numbers): Permits were obtained from Cape Nature (permit number: AAA004-00322-0035) and South African National Parks (permit: Agulhas and Table Mountain National Parks, issued 21 July 2010 to GJM).

### DNA Deposition

The following information was supplied regarding the deposition of DNA sequences: EMBL numbers are 16S: HG427449-51 ND2: HG427452-84.
